# “Exploring vertical task shifting: perceptions and experiences of nurses and general practitioners in Norwegian general practice - a qualitative study”

**DOI:** 10.1080/02813432.2026.2628664

**Published:** 2026-03-21

**Authors:** Marie Lovise Meland, Ona Rydningen, Ragnhild Bjarkøy Strandberg, Beate-Christin H. Kolltveit

**Affiliations:** Department of Health and Caring Sciences, Western Norway University of Applied Sciences, Haugesund, Norway

**Keywords:** Vertical task shifting, general practice, qualitative method, organizational structures, interdisciplinary teamwork, reflexive thematic analysis

## Abstract

**Objective:**

Task shifting between health care providers is seen as a strategy for combating increased demands in primary healthcare. This study explored the perceptions and experiences of general practitioners (GPs) and nurses regarding vertical task shifting in Norwegian general practice.

**Design and methods:**

We employed a qualitative design. Data were collected through semi-structured interviews with nurses (*n* = 6) and GPs (*n* = 5) across six municipalities in Norway. The data were analysed using reflexive thematic analysis.

**Results:**

The analysis resulted in two main themes, each with associated subthemes: 1) Trust plays a pivotal role in task shifting processes; (i) Time is essential for cultivating mutual trust, (ii) Increased knowledge and mutual respect strengthen trust in nurses competencies and responsibilities, and 2) Drivers and barriers to vertical task shifting; (i) Organizational structures influence utilization and perceived value of vertical task shifting, (ii) Choosing to work in interdisciplinary teams despite a lack of funding, (iii) Contextual factors and experience influenced attitudes towards vertical task shifting.

**Conclusion:**

Trust was described as pivotal to the success of vertical task shifting, with time and collaboration facilitating its development. In several cases, the process of task shifting expanded nurses’ roles and strengthened interdisciplinary relationships. However, organizational structures and funding significantly influenced the utilization and perception of task shifting. Attitudes toward task shifting varied based on individual and contextual factors.

## Introduction

Countries worldwide are experiencing significant demographic shifts and a shortage of skilled health personnel in primary healthcare, contributing to rising demand for healthcare services (1–3]. Organized at the municipal level, Norwegian primary healthcare encompasses a variety of services, including access to general practitioners (GPs). This “first line” of healthcare provides accessible, preventive, and curative services close to where people live, distinguishing it from “second line” specialist healthcare services [4, p. 44; 5]. According to [6], maintaining equitable access to healthcare remains challenging, largely due to the shortage of GPs. However, increased demand for primary care is not driven solely by shortages of GPs and other healthcare professionals. Rather, it is influenced by multiple factors, including population aging and the transfer of responsibilities from secondary to primary care. This redistribution is a key outcome of the Coordination Reform [7], which aimed to reduce hospitalizations, streamline patient care, and lower costs by shifting non-specialized services closer to patients. As a consequence, GPs have experienced a substantial increase in workload ([7]; 8, p. 25; 9, pp. 53–54).

National and international policy documents increasingly highlight task shifting as a strategy to address the growing demand for primary care services ([10,^11^]; 9,12, pp. 190–192; 13]. The World Health Organization [14] defines task shifting as the redistribution of specific tasks among health professionals, thereby introducing changes in established professional boundaries. Such reorganization is typically motivated by the need to use limited healthcare resources more efficiently. Task shifting may occur vertically – across professions with different educational levels – or horizontally – between professionals with comparable education [8,15].

Internationally, task shifting has been extensively studied both in primary health care and in outpatient clinics, and there is increasing advocacy for an interdisciplinary team-based approach as an alternative to single-profession general practices [3,16,17]. Studies demonstrate potential benefits, such as improved patient access and enhanced chronic disease management [18,19]. Norwegian primary healthcare is designed to provide accessible and comprehensive services; however, nurses have historically occupied a peripheral role within general practice, often undertaking administrative or medically adjacent tasks. In Norway, the most comprehensive and policy-driven attempt to operationalise vertical task shifting in general practice has been the Primary Health Care Team (PHCT) project (2018–2024). This pilot project introduced interdisciplinary teams consisting of GPs, nurses, and medical secretaries in 17 general practices across 9 of Norway’s 357 municipalities [9,20]. Through organizational and financial incentives, the PHCT model aimed to improve care for patients with complex needs. Early evaluations indicate positive effects on patient satisfaction and care coordination, while also raising concerns regarding the model’s scalability and transferability beyond specially supported settings [21].

Despite increasing policy support for interdisciplinary models of care, including the PHCT initiative, vertical task shifting continues to meet scepticism among Norwegian GPs. This is often found to be related to concerns about nurses’ competencies, fear of fragmentation of care, and the lack of stable structural and financial frameworks [22,23]. While trust, role clarity, and organizational flexibility are highlighted as key conditions for successful task shifting, these factors remain underexplored in Norwegian general practice settings [22,24].

Although national policy documents increasingly promote task shifting and more efficient resource use, implementation varies substantially across municipalities [25,26]. Implemented in a limited number of municipalities and general practices, the PHCT project has become the primary setting for empirical research on vertical task shifting in Norwegian general practice [8,21,27]. Consequently, existing evidence primarily reflects experiences from a narrow and specially supported subset of GP clinics, which may influence how vertical task shifting is currently understood within Norwegian general practice. To address this gap, the present study explores GPs and nurses’ perceptions and experiences of vertical task shifting across different organizational models, including PHCT clinics and traditional GP clinics.

## Materials and methods

### Study design

A qualitative design was chosen to gain a deeper understanding of how nurses and GPs perceive and experience vertical task shifting in Norwegian general practice. Our approach was inductive, using reflexive thematic analysis, as described by Braun and Clarke [28–31].

### Study context

In Norway, general practice is delivered through clinics that are either privately owned or municipally managed, funded by a combination of public resources and patient co-payments. All residents are entitled to a regular GP through the national Regular GP Scheme, which provides preventive, diagnostic, treatment, and follow-up services. Most GPs are self-employed contractors operating list-based practices (typically 1,000–1,200 patients per GP), usually in small clinics with 2–6 GPs supported by a few health secretaries and, less commonly, nurses. Funding combines capitation, fee-for-service (FFS), and patient co-payments, which tends to incentivize GP-led care over team-based approaches [9, 23]. Clinics following this traditional model are referred to here as *traditional clinics*.

In contrast, clinics within the PHCT project employed interdisciplinary teams comprising GPs, nurses, and health secretaries to enhance care for patients with complex needs through systematic, proactive, team-based follow-up. Nurses in PHCT clinics assumed expanded clinical roles, performing tasks traditionally carried out by GPs and additional responsibilities such as chronic disease management and comprehensive health assessments [20]. During the PHCT-project (2018–2024), nurses could also participate in the FFS system under their own billing codes, contributing directly to their salaries [21, 23]. These clinics are referred to as *PHCT clinics.*

### Settings and participants

We conducted our research across six municipalities in Southern and Mid-Norway, with populations ranging from <6,000 to >115,000. Using purposeful sampling, we contacted 11 general practices *via* email or phone and described the aim of our study. The inclusion criteria were registered nurses and GPs working in general practice, in both PHCT clinics and traditional clinics. Ultimately, 11 participants, comprising six GPs and five nurses across six general practices, chose to participate in the study (Table 1).

**Table 1.** Participants profession and context.

Participant	Profession	Context
1	Nurse	Traditional clinic
2	Nurse	Traditional clinic
3	GP	Traditional clinic
4	GP	Traditional clinic
5	GP	PHCT clinic
6	Nurse	PHCT clinic
7	Nurse	PHCT clinic
8	GP	Traditional clinic
9	Nurse	PHCT clinic
10	GP	PHCT clinic
11	GP	Traditional clinic

A combination of recruitment strategies was used. Some clinics were contacted through existing professional networks (e.g. previous clinical collaboration or professional acquaintance), while others – particularly PHCT clinics located elsewhere in the country – were identified through public evaluation reports from the PHCT project and contacted *via* email. The use of multiple recruitment pathways was necessary to secure variation in clinic type and location, and to obtain participants not restricted to the researchers’ immediate professional environment.

The participants had varied educational and training backgrounds. Among the nurses, two were nearing completion of a master’s programme to become a nurse practitioner, and several had completed additional courses in areas such as diabetes care, geriatrics, or wound management. Some of the nurses held bachelor’s degrees in nursing without formal post-graduate specialization. Nurses working in PHCT clinics had also received supervised clinical training as part of the project. Amongst the six GPs, four were specialists in family medicine. On average, the nurses had 16.6 years of clinical experience and the GPs had 17 years, calculated from the completion of their professional degrees.

### Data collection

We developed an interview guide (supplementary Table 1) and carried out individual semi-structured interviews in Norwegian. Before commencing data collection, a pilot interview was done to acquaint ourselves with the semi-structured guide and identify any necessary adjustments ahead of the data collection. Participants were provided with the option of either face-to-face or digital video interviews. For participants situated in different regions of the country, we exclusively utilized digital video link interviews. In local municipalities, all participants opted to meet in person. Between April 4 and June 25, 2024, a total of 11 interviews were completed and audio recorded. Averaging 21.6 min per interview, the audio recordings totaled 238 min. Both MLM and OR were present for 8 of the interviews. OR conducted one interview alone, whereas MLM conducted two interviews alone. Participants were asked to share their perceptions of the term vertical task shifting and their experiences of moving tasks from doctors to nurses, supported by the use of the semi-structured interview guide.

### Data analysis

We used reflexive thematic analysis (RTA) for our data analysis, following the six analytical steps outlined by Braun and Clarke [28], which focus on identifying patterns and themes within a given dataset. MLM and OR transcribed all audio recordings verbatim in Norwegian. The transcriptions were then organized by number, and reflexive thematic analysis was conducted manually with an inductive approach. MLM and OR read each transcript carefully and then proceeded to carry out the initial coding individually in keeping with the first step in RTA, which entails familiarization. Initially, the analysis began semantically, with MLM and OR generating codes explicitly based on participants’ descriptions. We then met to exchange and discuss thoughts and initial codes. Subsequently, our analysis evolved to more latent levels as we discussed and exchanged interpretations of meanings within the extracted data. This iterative process involved creating new codes through collaborative deliberation, clustering and pattern-searching, leading us to identify four candidate themes.

To unveil the core of each theme and ensure relevance, we collaboratively organized all codes and data extracts under corresponding potential themes. This process involved thorough discussions with RBS and BHK to align our findings with our overarching aim. Throughout this process, we engaged in constructive dialogue to facilitate further refinement, ultimately resulting in the identification of two main themes, each with two and three subthemes, respectively. The emphasis on teamwork throughout this process was crucial in enhancing the rigor and depth of the analysis.

Reflexive thematic analysis challenges traditional positivist perspectives by valuing the researcher’s subjectivity in the research process [31,32]. This approach emphasizes the significance of researcher reflexivity, which entails ongoing critical self-reflection throughout the research project [31,33]. Prior to commencing our study, we acknowledged that our beliefs and assumptions would influence our data interpretation. To enhance methodological rigor and trustworthiness, we followed Lincoln and Guba’s evaluative criteria—credibility, dependability, transferability, and confirmability [34]. Utilizing researcher crystallisation and maintaining thorough documentation allowed us to address credibility and dependability. Detailed descriptions of our findings, coupled with reflection on our preunderstandings, contributed to the transferability and confirmability of our study. All authors are registered nurses with experience in different primary care settings, mainly out-of-hours clinics, nursing homes and general practice.

### Ethical consideration

The study was approved by SIKT, the Norwegian Agency for Shared Services in Education and Research, on February 2, 2024 (Ref. no. 475567). Participants were provided with written and verbal information about the study and the principles of voluntary participation. Written consent was obtained before each audio recording, and participants had the option to withdraw at any time without providing a reason. Identifiable information was removed from transcripts, and all data was securely stored in accordance with Western Norway University of Applied Sciences’ guidelines.

## Results

The data analysis revealed two main themes with associated subthemes (Table 2).

**Table 2.** Themes and subthemes.

**Themes**	**Subthemes**
Trust plays a pivotal role in task shifting processes	Time is essential for cultivating mutual trust Increased knowledge and mutual respect strengthen trust in nurses’ competencies and responsibilities
**Drivers and barriers to vertical task shifting**	Organizational structures influence utilization and perceived value Choosing to work in interdisciplinary teams despite a lack of funding Contextual factors and experience influenced attitudes towards vertical task shifting

### Trust plays a pivotal role in task shifting processes

Our analysis indicates that trust is a critical factor for the effective establishment of new roles and responsibilities for nurses within general practice. Participants identified several contributing factors, including time, team-building initiatives, and the cultivation of mutual confidence in each other’s competencies, which will be further examined in the subthemes “time is essential for cultivating mutual trust” and “task shifting enhances confidence in nurses’ capabilities”.

#### Time is essential for cultivating mutual trust

Several participants encountered challenges in implementing vertical task shifting, with time frequently identified as a crucial determinant of success. Within the context of the PHCT-project, both GPs and nurses emphasized the importance of adequate time allocation for establishing a sense of security and trust in their new roles and responsibilities. One nurse, serving in a GP-led PHCT clinic, explained that her first year was vital for her professional growth, stating that she was given time to navigate through her inexperience under the close guidance of doctors. Similarly, GPs working in PHCTs cited the initial implementation phase – when the clinic transitioned to team-based care by shifting tasks from doctors to nurses – as a critical period for building trust within a new model for patient care. For example, members of the PHCT-project were given time to get acquainted with one another personally and professionally through fully funded team-building exercises and seminars. As noted by one participant:

We invested a lot of time in team-building exercises at the outset. We had to collectively step out of our comfort zones and as a result, we became more confident in each other. (Participant 7)

Participants stressed the importance of reducing their professional barriers through engaging in seminar activities unrelated to work. They expressed the importance of having time to become familiar with each other’s strengths and weaknesses in a low-stakes environment. Upon returning to their professional roles, they had already navigated awkward situations and resolved problems under stress. Several participants reported enhanced collaboration and mutual trust as a consequence of this training.

Participants not involved in the PHCT-project emphasized the importance of allocating sufficient time during the initial stages of implementing vertical task shifting. One GP discussed their decision to prioritize resources to employ a nurse to manage follow-up care for diabetes patients, stating:

I have given this some thought; for it [task shifting] to succeed, it requires a nurse who is eager to advance her education and a doctor who can dedicate adequate time for training and collaboration. (Participant 3)

Moreover, participants in PHCT clinics highlighted the importance of making time for routinely scheduled meetings between nurses and doctors to review patient cases, which served as a mechanism for quality control. Several participating nurses noted the need for sufficient time to familiarize themselves with patients’ medical records and test results in collaboration with GPs. These formal and informal meeting points between nurses and physicians also helped enhance trust in each other as equal members of the team.

GPs involved in the PHCT project stressed the need for comprehensive training, guidance, and clear communication to build trust and facilitate successful task shifting from GPs to nurses, which is a time-consuming process. Nurses emphasized the security provided by permanent staff over time and the trust built through long-term collaboration. However, they also acknowledge that preexisting power structures can pose a challenge. One nurse elaborated on her experience of being new to the team:

It was a little difficult to know what my limits were. Like, what did I have permission to do independently? […] I had to phrase my questions carefully, like, ‘would it be smart do this or that’, or; ‘what are your thoughts on X’. Phrase it in a way that revealed my knowledge on the subject without straight up telling the doctor ‘this is what we should do’. Because the doctors want to be the ones in charge of treatment, as they should. […] Every doctor is different, and I had to learn their ways and, in time, gain their trust (Participant 6)

#### Increased knowledge and mutual respect strengthen trust in nurses’ competencies and responsibilities

Our analysis revealed the significance of recognizing each other’s professional competencies as essential for fostering trust between GPs and nurses. Multiple GPs acknowledged that their familiarity with nurses’ clinical assessment skills significantly boosted their trust in them. Moreover, participants who experienced a systematic implementation of task shifting through the PHCT project indicated greater confidence in the range of functions nurses are equipped to manage. Our findings also suggest that the evolution of nurses’ responsibilities occurred in tandem with the process of trust-building among physicians. One participant remarked:

Confidence is a result of getting to know the level of each other’s competence. A reciprocal relationship of trust is important. (Participant 1)

One GP emphasized that knowing each other’s expertise fosters trust, leading to greater confidence in delegating tasks. Another GP described the evolution of their nurse’s responsibilities from protocol and lab work to conducting clinical assessments, including anamnesis, clinical examination, and proper documentation. Both GPs and nurses stated the importance of knowing one’s own limits and not being overconfident. A GP articulated this:

When I have full confidence in the nurse, I take a step back. […] When you work closely together over time, you constantly develop trust in each other’s competencies. (Participant 3)

Nurses within the PHCT-project also discussed being able to make home visits to patients unable to visit the doctor’s office. They talked about conducting comprehensive assessments, communicating findings to the GP, and preparing patients for hospital admission if necessary. Home visits by a general practice nurse were exclusively conducted within the PHCT setting. None of the participants outside of the PHCT-project had implemented such independent roles for their nurses. Some PHCT nurses reported that their evolving tasks and responsibilities stemmed from a systematic approach to task shifting, developed in close collaboration with GPs. This process fostered mutual trust, enhancing GPs’ confidence in the nurses’ clinical assessment abilities. One GP in a PHCT elaborated:

Well, we doctors are traditionally fond of maintaining full responsibility for the ‘big picture’ of our patients’ treatment and care. […] We have had to work extensively to accept that nurses bear their own responsibilities for consultations and documentation. […] This has perhaps been the most significant hurdle [in implementing task shifting]. To kind of get my colleagues in on the idea that yes, nurses do make mistakes, but they are also accountable for those mistakes. […] As competence increases, so too does responsibility, right? (Participant 5)

A GP from another PHCT elaborated on this subject by explaining how tasks are delegated based on the competencies of each healthcare professional within the PHCT. Responsibilities such as laboratory work and vital sign collection were reserved for health secretaries, while tasks requiring consultation and clinical assessment experience were assigned to nurses. Over time, systematic task shifting led to an expansion of nurses’ responsibilities. This participant remarked:

We adhere to the LEON principle: lowest effective level of care. Patients who are eligible will be assessed and treated by a nurse. If a secretary can perform the task, they will handle it instead. […] The nurse in our team is currently pursuing a master’s degree to become a nurse practitioner. She is learning to examine patients in a more advanced manner, similar to doctors. Consequently, we are working to expand her responsibilities, for instance, by scheduling patients with upper airway infections on her list. […] Eventually, we aspire for nurse practitioners to have the authority to prescribe certain medications. (Participant 10)

GPs without formal task shifting experience, serving in traditional clinic models, also acknowledged the importance of collaborating with nurses and understanding task shifting concepts for effective teamwork and patient care. However, opinions on which tasks nurses could undertake differed significantly from participants within PHCTs. One GP expressed concerns:

I find it acceptable for the nurse to collect vital signs, CRP levels, urine samples, and similar tasks. If they are capable of doing these tasks, that is completely fine with me. […] It’s acceptable for the nurse to assist the doctor, but that assistance must be offered with caution. The nurse has to weigh their words carefully. Avoid being authoritarian and too assertive. At the end of the day, the responsibility rests with the doctor. (Participant 8)

This statement reveals a discrepancy between the advanced tasks GPs within PHCTs deem appropriate for nurses in general practice and the more basic nursing skills outlined by those in traditional general practice settings. A majority of participants, especially those involved in the PHCT-project, described task shifting as a dynamic and continually evolving process. This process itself seemed to enhance GPs’ confidence in nurses’ competencies as physicians gradually learned to trust and acknowledge nurses’ skills through guided collaboration.

### Drivers and barriers to vertical task shifting

This theme examines motivators and barriers to shifting tasks from GPs to nurses in general practice. The data reveals how participants perceive the importance of external supports, such as organizational and economic frameworks, alongside internal influences, such as attitudes. It also highlights the strategies participants employ to navigate and overcome these challenges.

#### Organizational structures influence utilization and perceived value

Nurses working in PHCT clinics reported having their own billing rates, enabling them to see patients independently without GP involvement unless necessary. Conversely, nurses outside the PHCT-project lacked this opportunity, citing organizational structure as a major barrier to task shifting. One GP expressed his desire for his clinic to incorporate task shifting to various healthcare professionals and create a collaborative, multidisciplinary team. However, he noted that the existing organizational framework makes this transition difficult and costly. He mentioned that although they have hired nurses to help manage patients with diabetes and COPD, he had to attend every nurse consultation to be able to bill for their services. When asked about his thoughts on profession-neutral billing rates, he said:

Oh, I would gladly retreat. […] I have full confidence in the nurses working here and would have had no reservations about [name of nurse] conducting consultations on her own. I trust she recognizes when to seek assistance. (Participant 3)

The capacity to bill for completed work was expressed by both nurses and GPs as an integral motivational driver for vertical task shifting. While only participants from PHCTs had practical experience with nurses’ billing rates, many outside the project expressed a strong interest in this capability. A unique aspect for participants involved in the PHCT-project was assessing when to consult with the patient’s GP. As one participant explained:

If I see twelve patients in a day, I might consult a doctor about four of them to confirm my findings. (Participant 10)

The Primary Healthcare Team project was set to end in June 2024, and with it, the nurses’ billing rates, which prompted reflections from several participants about the future of interdisciplinary teamwork in Norwegian general practice. PHCT nurses voiced feelings of uncertainty and fear for their jobs, as the project’s termination would mean losing their ability to bill for their services and thus generate income. A PHCT physician stated that person-centred care might be harder to maintain if they lose the nurses:

As a PHCT, we have primarily focused on patient populations who may struggle to visit a doctor’s office or possess low health literacy, such as individuals with disabilities and the elderly. It is… quite challenging for a GP to maintain comprehensive oversight in these complex cases, particularly when adherence to treatment is inconsistent. This is where we see the biggest gain from involving PHCT nurses, who can conduct home visits, perform bloodwork in the patient’s home and so on. This facilitates a far more thorough follow-up. […] When the project ends, these patients will be the biggest losers. (Participant 5)

Participants working in conventional clinic settings lacked access to the organizational advantages afforded by the PHCT-project, such as the ability to utilize nurses’ billing rates and having a dedicated PHCT leader. Nonetheless, some GP participants still employed nurses, albeit in a modified role. These nurses reported their primary tasks as administrative work, answering phone calls, triage and lab work. They also experienced some varying degrees of task shifting from GPs, mainly diabetes and COPD follow-ups. This required time to familiarize themselves with patients’ latest bloodwork and medical histories, leading them to step away from other clinical duties and phone calls, which sometimes led to a sense of being understaffed, as described by one nurse:

I’m not able to schedule a full day of consultations with COPD patients. If I could, I’d have a better workflow and understanding of my patients. Right now, I spend time answering phones, assisting in the lab, and before I know it, I’m behind schedule and don’t have the chance to prepare. (Participant 2)

Overall, the organizational context in which participants operated, specifically whether they were part of a PHCT or a traditional clinic model, significantly influenced their degree of utilization and their perceptions of task shifting.

#### Choosing to work in interdisciplinary teams despite a lack of funding

Participants in the PHCT-project reported receiving government funding, which helped facilitate the employment of nurses. This contrasted with GPs in traditional clinics, who noted that they had to bear the cost of nurses’ salaries without access to financial support. Notably, two participating GPs explained that they opted to hire nurses despite their ineligibility for financial assistance, emphasizing the benefits of sharing patient loads with nursing staff. One physician also articulated a desire to shift the financial burden associated with nurses’ pension rights to local municipalities. Municipal support was cited by numerous participants as important in advancing a more team-oriented approach within general practice.

A GP elaborated on this, expressing concern over the current economic models:

No provision has been made for this [task shifting]. […] The tasks we allocate the nurses… We have to pay for it ourselves, and we get no money in return. (Participant 4)

As the PHCT-project approached its conclusion, several participating GPs conveyed apprehension about having to cover nurses’ salaries out of their own pockets. Despite economic challenges, participants demonstrated a sense of responsibility within their teams, recognizing that the clinic had become reliant on nurses as a vital resource. Both nurses and doctors reported receiving positive feedback from patients, who valued the additional time nurses dedicated to gathering information that may otherwise have been overlooked. Nurses reported that providing comprehensive care contributing to health promotion and increased health literacy were significant aspects of their role in general practice. One GP agreed, stating:

I believe everyone feels a sense of responsibility for the project and that each person is equally important for the team’s success. We are focusing more on prevention and are able to detect diseases earlier. […] But obviously, it’s all about money in the end. (Participant 10)

Four participating GPs from both organizational frameworks expressed their willingness to continue employing nurses; however, they noted that this would present a financial challenge moving forward, making task shifting difficult. One GP put it this way:

It certainly provides added value to have nurses in the doctor’s office, and I cannot imagine how we will work and maintain the same quality without them. I feel that everything has worked well. […] It is the financing that is the challenge now. (Participant 8)

#### Contextual factors and experience influenced attitudes towards vertical task shifting

GPs with experience in task shifting generally reported positive attitudes toward this approach. Although they typically expressed satisfaction with their roles as GPs, they occasionally felt overworked. Nurses in both PHCT and traditional clinics also reported a positive outlook on vertical task shifting and expressed being able to alleviate physicians from some of their responsibilities. Nevertheless, nurses also voiced a desire to further develop the roles of nurses within general practice and indicated a wish for greater autonomy in their patient consultations.

Multiple participating GPs emphasized that collaboration with nurses and the involvement of professionals with diverse academic backgrounds helped enhance patient safety. However, they observed that delegating additional tasks to nurses did not necessarily reduce their overall workload; instead, it changed the nature of their tasks. The quality assurance provided by nurses was viewed positively, alleviating mental strain and making it possible to spend more time on complex cases. However, this was not always the case, as some participants described a somewhat hierarchal structure at the start of the PHCT-project. One PHCT physician stated:

In the beginning of the [PHCT] project, our doctors were very afraid that diagnostics or treatment would be delayed or go wrong because of the active involvement of nurses. This has been our most significant challenge to overcome. That’s why it is crucial to have a project leader who can repeatedly emphasize that nurses bear their own weight of responsibility in this. (Participant 5)

This participant described the challenge of clarifying responsibilities and the fear that errors may occur when tasks are shifted. Consequently, the PHCT team leader, possessing knowledge of relevant laws and regulations, was described as essential in providing clear guidance on which practices were safe and permissible within healthcare legislation. With the legal framework established, physicians articulated their personal attitudes towards task shifting gradually transitioned to a more opportunity-focused perspective. One GP summed this up:

We are used to having the last word for better or worse, I think. Task shifting challenges that. (Participant 11).

Primary care physicians with limited experience in vertical task shifting emphasized the need for clear communication and mutual understanding of task shifting concepts, prioritizing patient benefit. Concerns about GPs losing touch with their patients if nurses took over specific tasks were mentioned, though some viewed this fear as unfounded. Perceptions of task shifting varied notably depending on involvement in the PHCT-project. Those outside the project often worried about missing crucial patient information and bearing responsibility for errors made by nurses. Conversely, a PHCT physician shared a more optimistic perspective. When asked about potential risks of transferring tasks from doctors to nurses in general practice, he responded:

Well, when we compare general practice to hospitals and outpatient clinics, why should we operate in a fundamentally different way? They [hospitals and outpatient clinics] successfully utilize nurses in close collaboration with doctors, so why shouldn’t we? In my opinion, task shifting benefits the patient and alleviates the workload for GPs. (Participant 5)

Overall, our findings revealed a spectrum of attitudes toward vertical task shifting, shaped by participants’ organizational contexts and prior experiences. The majority of participants underlined the importance of keeping the patient’s best interests in mind during the process of task shifting. PHCT physicians generally displayed a more open-minded approach, while those with limited exposure to task shifting tended to be more cautious. Consequently, attitudes can serve as both a motivational driver and a barrier to the successful implementation of task shifting in general practice.

## Discussion

### Summary of main results

Our study’s findings focused on two main themes: the crucial role of trust in the task shifting process and the various organizational drivers and barriers associated with it. Both GPs and nurses recognized task shifting as a national strategy aimed at optimizing resource use. However, their perceptions and experiences differed based on their specific organizational contexts. Whitin the PHCT-project, participants worked systematically to implement a vertical shift of tasks from GPs to nurses and displayed predominantly positive attitudes towards this approach. In contrast, participants from traditional clinics displayed varying degrees of engagement with task shifting, and not all of them recognized the need for nurses in addition to health secretaries in general practice.

### Comparison with existing literature and the role of trust

Consistent with findings from previous studies [20,21,35,36], our research revealed that both GPs and nurses identified trust as a crucial determinant for the successful implementation of vertical task shifting within their teams. Participants from both PHCTs and traditional clinics emphasized trust as a collaborative and evolving process essential for the effective integration of nurses into general practice. Notably, while both GPs and nurses acknowledged the significance of trust, the discourse predominantly focused on the trust that GPs placed in nurses rather than the reciprocal trust from nurses towards GPs. Our results indicated that nurses had to adapt to the different personality traits of the GPs and used deliberate communication strategies to gain their trust. Similar findings have been reported [37,38], highlighting that hierarchy and traditional power structures were challenged by new collaborative models of primary care.

In this study, interprofessional trust barriers were most prominent among participants with limited experience in vertical task shifting. This aligns with evaluations of the PHCT project, which noted challenges in building trust between GPs and nurses one year into the project, particularly in clinics that had not previously employed nurses [20]. These findings are consistent with research that states the importance of having a clear vision and mission statement endorsed by all members of the general practice team when implementing skill mix into clinics [39,40]. However, our findings suggest that GPs in traditional clinics displayed some hesitance regarding which tasks and responsibilities were suitable for nurses. This scepticism may be linked to these participants’ limited exposure to interdisciplinary teamwork in a general practice setting. Prior research have emphasized that sustained collaboration amond healthcare professionals were crucial for fostering trust, which in turn enhanced team performance [41,42]. Our study supports this notion, indicating that as GPs gained experience and increased their understanding of task shifting, their confidence in nurses’ undertaking responsibilities within their competencies and legal parameters increased.

### Drivers, barriers and patient safety concerns

In our study we found that participants in the PHCT-project perceived task shifting as a means to reduce GPs workload and better patient care by introducing a more holistic approach. Research show that integrated care models that prioritize collaborative practice among healthcare providers can enhance patient outcomes, reduce unnecessary hospitalizations and improve staff morale [43–46]. In keeping with existing research [21], we found that GPs who had implemented task shifting did not experience a dramatic reduction in work tasks, but reported having more time for quality improvement. The PHCT-project enabled nurses to operate somewhat independently by having dedicated billing rates. Our findings suggests that this autonomy improved job satisfaction among PHCT GPs and nurses. Conversely, nurses and some GPs outside the PHCT framework expressed frustration at the limitations imposed by rigid organizational structures, which hindered their ability to engage fully in task shifting. They described lacking organizational and financial support structures, revealing a gap that could impede the efficient use of nurses. One nurse described feeling undermined by administrative responsibilities instead of being able to focus on patient consultations. These observations resonate with existing literature that highlights the importance of supportive organizational mechanisms in facilitating effective task shifting [9,47]. Nonetheless, our study found that some GPs still opted to employ nurses and attempted to facilitate task shifting in their clinics despite the lack of organizational and financial support.

Contrasting viewpoints exist in the literature regarding the effectiveness and safety of task shifting. Some studies [8,48] suggest that broader implementation of task shifting may lead to inconsistencies in care delivery and reduced quality of patient interactions, potentially increasing the risk of role confusion and miscommunication in clinical settings. One study examining horizontal task shifting from specialised hospital doctors to GPs reported concerns related to patient safety [8]. Although this study focused on a different context, some of its findings may still be applicable to our results, as several GPs in our study reported fear of misdiagnosis or mistreatment as a significant barrier to reallocating responsibilities and tasks to nurses.

### Interprofessional boundary work and policy implications

Our study identified a disparity between participants in the PHCT project and those working in conventional clinics concerning their attitudes towards incorporating nurses into general practice and the implications for patient safety. PHCT participants valued the flexibility afforded by nurses conducting home visits and performing follow-up care. In contrast, GPs in traditional clinics tended to take a more cautious stance. While some were open to the idea of integrating nurses into GP clinics, they emphasized the need to prioritize patient interests. At the same time, nurses reflected on being met with doubt and critical attitudes from some GPs, describing this as a significant barrier to their work. The GPs’ scepticism seemed rooted in the valid concern that adding more healthcare professionals to the clinics could lead to fragmented care and reduced patient continuity. Research have found that some GPs believe that delegating home visits to nurses may pose a risk to patient safety [49]. However, other studies emphasize that safe task shifting depends on clear leadership and adequate resources, particularly the presence of a team leader, while still upholding the GPs’ overall medical responsibility [50,51]. Understanding the scope of practice and limitations of nurses was viewed as critical to avoid unfortunate task shifting [52]. This divergence in perspectives highlights the need for tailored approaches that take into account the specific organizational contexts in which task shifting is implemented.

While this study shows that vertical task shifting in general practice presented several benefits, it also introduced challenges related to interdisciplinary boundary work, with participating GPs and nurses highlighting that the negotiation of roles between GPs and nurses were critical to maintaining effective collaboration and ensuring patient safety. One study found that ambiguous role definitions and poor communication were central challenges to interdisciplinary collaboration [53]. They emphasize that these barriers were particularly significant in primary care settings, where GPs and other professionals may be less accustomed to a team-based approach. If the delineation between tasks is unclear, it may result in overlapping responsibilities that can hinder effectiveness and lead to adverse patient outcomes [48,54]. Eliassen and Moholdt [54] identified two practices in their study of boundary work in task-shifting processes: “the engine and the assistant” and “the symbiotic team.” The first represents a hierarchical structure with clear role separation, while the second described a more collaborative, flat team dynamic in which all professions shared responsibilities and were expected to work together despite different academic backgrounds. Our findings suggest that participants in PHCTs tended to align more with Eliassen and Moholdts’ [54] idea of a symbiotic team characterized by higher levels of nurse autonomy, GPs’ trust in nurses’ capabilities, and opportunity-oriented attitudes. In contrast, participants in traditional clinics more closely fit the “engine and the assistant” model, with some GPs expressing limited perspectives on nurses’ roles in general practice. Another dimension highlighted by PHCT nurses and GPs was the termination of the PHCT project, which raised concerns about sustaining interdisciplinary collaboration beyond the project’s lifespan. The expressed fears regarding job security and the loss of financing reflected broader issues in healthcare policy [9,21], and mirrored predictions made by participants regarding the repercussions for vulnerable patient populations once the PHCT billing and financial support was withdrawn. The potential weakening of care for weak demanders, those with low health literacy or complex health challenges, accentuated the need for systemic solutions that can maintain the contributions of nursing staff within a collaborative team. The crux of the debate reflects the balance between incentivizing task shifting and maintaining safe, high-quality healthcare.

### Implications for practice and future research

Overall, our findings suggest that vertical task shifting in general practice presents meaningful opportunities to enhance care delivery, strengthen interdisciplinary collaboration, and alleviate pressures on GPs when supported by clear organizational frameworks. However, persistent concerns related to trust, role clarity, and patient safety underscore the need for systematic approaches that include adequate funding, leadership support, and shared responsibility. Ensuring sustainable integration of nurses into Norwegian general practice will require policies that recognize the contributions of nursing staff, promote collaborative team cultures, while protecting quality of care. Future research should examine longer-term effects once project-based funding for the PHCT-project has ended, include patient perspectives, and further explore how interdisciplinary boundary work can be managed across varying organizational models of GP clinics. More research is needed to explore the scope of nurses’ roles and vertical task shifting in various organizational contexts within Norwegian general practice.

## Strengths and limitations

All interviews were conducted in Norwegian, and the dataset was analysed in its original language. The results were then written directly in English. This approach follows standard qualitative research practice, where data are collected in participants’ native language to capture nuance and contextual meaning, and subsequently translated for reporting. Writing the results directly in English from a Norwegian dataset may have led to subtle shifts in nuance or interpretation, while the translation of selected participant quotes could also introduce minor discrepancies. However, care was taken to preserve the clarity, accuracy, and intent of participants’ accounts throughout this process.

Our study aimed to achieve methodological rigour by adhering to Lincoln’s and Guba’s [55] four evaluative criteria of dependability, transferability, credibility and confirmability. This study’s strengths lie in its broad inclusion criteria, which allowed for rich variation in data from interviews with GPs and nurses in clinics of various organizational structures across multiple municipalities. However, this variation meant that not all participants had experience with vertical task shifting in a general practice setting, which could potentially limit transferability of the findings. Nonetheless, we discovered that participants with limited experience in reallocating tasks to nurses had perceptions and insights regarding task shifting that could shed light on how to develop appropriate nursing roles in general practice. Transferability of our findings is most applicable to primary care systems with organizational models comparable to Norwegian general practice; however, findings concerning trust and evolving nursing roles may extend to international contexts undergoing similar shifts in professional responsibilities.

Several interviews were conducted *via* video links, which might have affected rapport and trust between interviewers and interviewees. However, research has indicated that video link interviews in qualitative studies also have advantages. They can broaden participant inclusion by removing logistical barriers, thereby enhancing the richness of data collection [56].

Our findings reveal a variation in attitudes toward vertical task shifting between participants in PHCT clinics and those in traditional clinics. As noted by [20] participation in the PHCT project was voluntary, attracting GP clinics interested in testing new care models. Consequently, participants from PHCT clinics may have approached the concept of task shifting with a more positive outlook from the outset. At the point of data collection, the PHCT clinics had additional staffing and financial support, offering a more favourable environment for task shifting. To address potential bias and ensure variation in data, our study intentionally included GPs and nurses from traditional clinics.

All four authors share a professional background in nursing, though with experience from different clinical settings. This may have influenced study design, participant engagement, and the interpretation of findings. Although this shared perspective presents a potential source of bias, we actively engaged in reflexive discussions throughout the research process to remain aware of how our positions might shape the analysis. Reflexivity involves critically examining how researchers’ backgrounds and contexts influence the conduct and interpretation of qualitative research [33].

## Conclusion

Our findings offer insights into how GPs and nurses in Norwegian general practice perceived and experienced vertical task shifting. Participants identified trust as a key factor for implementing task shifting, emphasizing that its development relies on time and sustained collaboration. In several instances, the task shifting process was described as a means to expand nurses’ roles and strengthen interdisciplinary relationships. Additionally, task shifting was stated to enhance quality and patient safety. However, organisational and financial structures influence the extent to which task shifting is adopted and valued. Furthermore, internal factors, such as attitudes toward task shifting, are shaped by individual experiences and organizational factors. Our findings underscore the necessity for robust organizational and financial support systems to enable the effective implementation of task shifting.

## Supplementary Material

Supplemental Material

## Data Availability

Our dataset is not available due to legal and ethical restrictions.
