# Supplementary material for: “Exploring vertical task shifting: perceptions and experiences of nurses and general practitioners in Norwegian general practice - a qualitative study”
Source: Scand J Prim Health Care. 2026 Mar 21;44(1):2628664. doi: 10.1080/02813432.2026.2628664 (PMC13007398; doi:10.1080/02813432.2026.2628664)
Supplement: Supplemental Material [file IPRI_A_2628664_SM6518.docx]

Supplementary table 1 – Interview Guide

"Exploring Vertical Task Shifting: Experiences and perceptions of general practitioners and nurses in Norwegian general practice"

| Opening Questions:      What is your title and position?    How long have you been working as a GP/nurse?    Can you tell me about your current work tasks?    Are there some tasks or responsibilities that are particularly time-consuming? | Elaborative questions:  What is your formal role at the clinic?    How do you perceive your role at the doctor's office?    How long have you been in this role?    What are your thoughts about your current role?    How would you like your role to be structured?    Anything you would like to change?    Has there been any discussion in the workplace about professional roles and/or boundaries? |
| --- | --- |
| Main Questions:    How do you understand the term task shifting?  What is your experience with vertical task shifting in a general practice setting?    What are your thoughts on how to ensure successful task shifting from doctors to nurses?    Do you have any thoughts on what might be lost or at risk when delegating tasks to professionals with lower formal qualifications? | *If the participant has experience with task shifting*:  Can you specify how task shifting has been implemented at your workplace? Which tasks have been redistributed, from whom and to whom, etc.  What is your experience working in a multidisciplinary/team-based setting in general practice? |
| Closing Questions:    Is there anything we haven't covered in this conversation that you would like to highlight or talk about? |  |
